# A pilot study using dynamic contrast enhanced-MRI as a response biomarker of the radioprotective effect of memantine in patients receiving whole brain radiotherapy

**DOI:** 10.18632/oncotarget.9653

**Published:** 2016-05-28

**Authors:** Philip Wong, Ilana R. Leppert, David Roberge, Karim Boudam, Paul D. Brown, Thierry Muanza, G. Bruce Pike, Jeffrey Chankowsky, Catalin Mihalcioiu

**Affiliations:** ^1^ Department of Radiation Oncology, Centre Hospitalier de L'Université de Montréal, Montréal, Québec, Canada; ^2^ Montreal Neurological Institute, McGill University, Montreal, Quebec, Canada; ^3^ Department of Radiation Oncology, Division of Radiation Oncology, the University of Texas MD Anderson Cancer Center, Houston, TX, United States of America; ^4^ Department of Oncology, Jewish General Hospital, Montreal, Québec, Canada; ^5^ Departments of Radiology and Clinical Neurosciences, Hotchkiss Brain Institute, University of Calgary, Alberta, Canada; ^6^ Diagnostic Radiology, McGill University Health Center, Montréal, Québec, Canada; ^7^ Department of Oncology, McGill University Health Center, Montreal, Québec, Canada

**Keywords:** MRI, biomarker, imaging, radiotherapy, memantine

## Abstract

**Purpose:**

This pilot prospective study sought to determine whether dynamic contrast enhanced MRI (DCE-MRI) could be used as a clinical imaging biomarker of tissue toxicity from whole brain radiotherapy (WBRT).

**Method:**

14 patients who received WBRT were imaged using dynamic contrast enhanced DCE-MRI prior to and at 8-weeks, 16-weeks and 24-weeks after the initiation of WBRT. Twelve of the patients were also enrolled in the RTOG 0614 trial, which randomized patients to the use of placebo or memantine. After the unblinding of the treatments received by RTOG 0614 patients, DCE-MRI measures of tumor tissue and normal appearing white matter (NAWM) vascular permeability (Initial Area Under the Curve (AUC) Blood Adjusted) was analyzed. Cognitive, quality-of-life (QOL) assessment and blood samples were collected according to the patient's ability to tolerate the exams. Circulating endothelial cells (CEC) were measured using flow cytometry.

**Results:**

Following WBRT, there was an increasing trend in the vascular permeability of tumors (*p*=0.09) and NAWM (*p*=0.06) with time. Memantine significantly (*p*=0.01) reduced NAWM AUC changes following radiotherapy. Patients on memantine retained (COWA *p*= 0.03) better cognitive functions than those on placebo. No association was observed between the level of CEC and DCE-MRI changes, time from radiotherapy or memantine use.

**Conclusions:**

DCE-MRI can detect vascular damage secondary to WBRT. Our data suggests that memantine reduces WBRT-induced brain vasculature damages.

## INTRODUCTION

The incidence of brain metastasis in cancer patients has been 30-40% [1–3]. This figure may rise as novel therapeutic agents improve the systemic control of cancers outside of the central nervous system. The brain represents a watershed area for tumor metastasis to seed and colonize as the normal blood-brain-barrier (BBB) prevents systemic agents from reaching tumor cells. Radiotherapy currently remains as the main treatment option for patients with brain metastases [4].

Despite the increasing use of radiosurgery to treat brain metastases, patients with brain metastases often receive whole brain irradiation (WBRT) for widespread metastases, post-radiosurgery progression or leptomeningeal seeding [4]. WBRT may control the progression of brain metastases and lengthen lifespan, but it may induce cognitive deficits. As radiation may induce neuronal N-methy-D-aspartate (NMDA) receptor stimulation and excitotoxicity, inhibition of the NMDA receptor using a competitive antagonist, memantine may reduce WBRT-induced cognitive decline. The Radiation Therapy Oncology Group (RTOG) 0614 study randomized patients treated with WBRT to receive concurrent placebo or memantine [5]. The RTOG 0614 study demonstrated that patients who received memantine maintained better cognitive functions than patients who had placebo [5].

*In vivo* murine experiments suggested that the side-effects of radiotherapy to normal brain tissue is secondary to the induction of global vascular damages in the form of devascularization, gliosis, demyelination and white matter necrosis [6]. A preliminary clinical study suggested that Dynamic Contrast-Enhanced MRI (DCE-MRI) could detect increased permeability of the BBB and blood-tumor-barrier at the completion of 60Gy of fractioned radiotherapy for the treatment of glioma [7]. Similarly, it has been suggested that DCE-MRI could be used to assess the efficacy of focused ultrasound in disrupting the BBB [8].

The current pilot study aims at determining whether DCE-MRI is able to detect and measure changes in vascular permeability during the first 6 months following WBRT (37.5 Gy in 15 fractions) in patients with brain metastasis, and whether vascular changes in normal appearing white matter (NAWM) are associated with neurocognitive function and/or memantine use.

## RESULTS

Our patient population was composed of a heterogeneous group of patients with different cancer histology and who had received various local treatments prior to WBRT (see Table 1). Most patients had lung (*n* = 7) or breast cancers (*n* = 4). Twelve patients had unresected tumor(s) present in the brain at the time of WBRT. The median overall survival from WBRT initiation was 9.75 (0-33.6) months. Seven patients received memantine, while 7 patients received either placebo or neither.

**Table 1:** Characteristics of the study patients

Pt #	Primary disease	Age at time of WBRT	Number of brain lesions	Volume of tumor analyzed (cc)	Time between WBRT and prior surgery or SRS brain treatment	OS from WBRT (months)	Use of Pla, Mem or Neither
1	Breast	34	1	1.73	Sx: 4 weeks	20.5	Pla
2	Breast	53	4	0.78	N/A	33.6	Pla
3	Lung	71	2	0.62	Sx: 6 weeks RS: 4 weeks	23.4	Neither
4	Breast	51	1	1.58	Sx: 6 weeks RS: 4 weeks	18.6	Mem
5	Colon	66	4	0.48	RS: 4 weeks	4.0	Mem
6	Lung	65	>10	0.94	N/A	5.6	Mem
7	Lung	66	4	13.06	N/A	10.9	Mem
8	Melanoma	65	3	0.53	RS: 5 weeks	4.9	Mem
9	Thryoid	57	2	1.78	RS: 59 weeks	21	Neither
10	Lung	67	6	0.60	N/A	7.2	Plac
11	Lung	44	1	3.70	RS: 2 weeks	0	Mem
12	Lung	78	>10	0.62	N/A	5.9	Plac
13	Lung	63	2	1.37	Sx: 5 weeks RS: 2 weeks	28.5	Mem
14	Breast	57	2	0.65	RS: 44 weeks	8.6	Plac

WBRT: Whole brain radiotherapy; Sx: Surgery; RS: Radiosurgery; N/A: no surgery or RS; OS: Overall Survival; Pla: Placebo; Mem: Memantine

**Figure 1:**
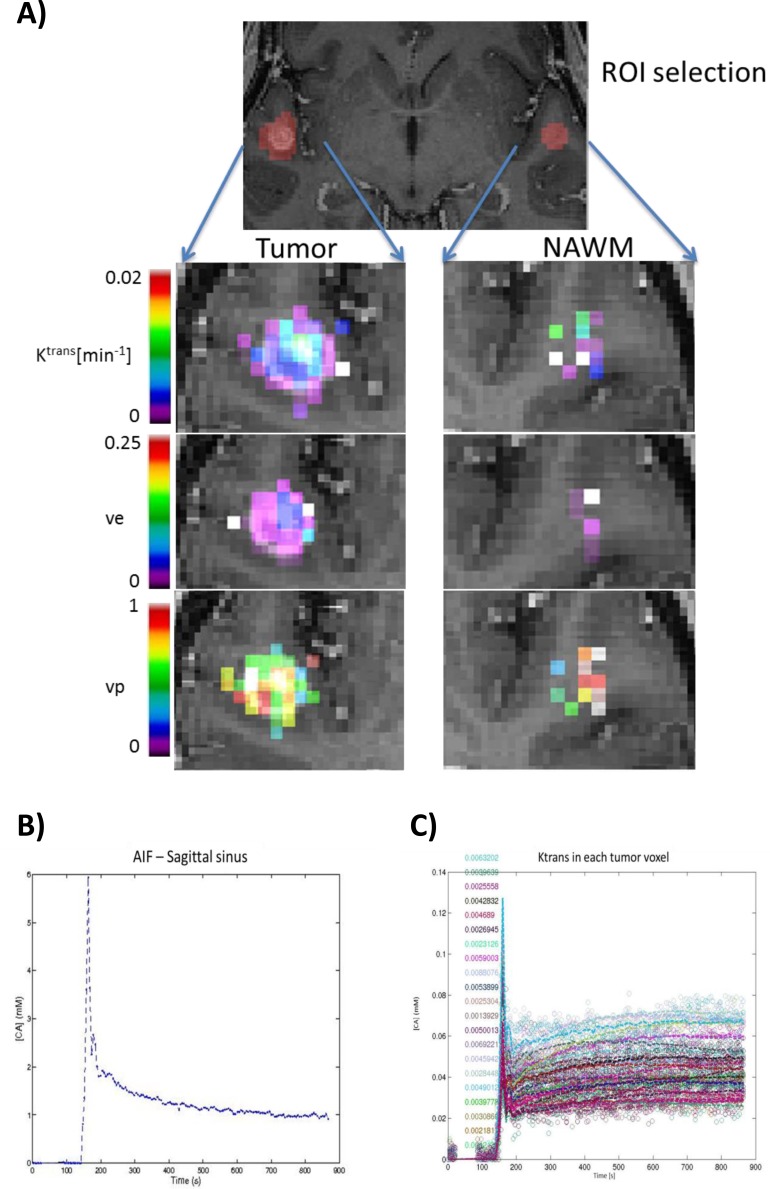
Sample parametric maps and time course of a tumor Using the Tofts and Kermode modeling of DCE-MRI data, **A.** parametric maps of Ktrans, ve and vp were obtained for Tumor and normal appearing white matter (NAWM). Note that when the fit did not converge (Ra2 < 50%), all the parameters are set to 0. This was the case for many voxels within the region of interest (ROI) defined as NAWM. Ktrans: transfer constant; ve: extravascular extracellular space (EES) fractional volume; vp: blood plasma volume. A sample time course of the constrast agent concentration [CA] within **B.** the sagittal sinus (AIF) and **C.** each Tumor voxel from Figure 1A.

Compared to K^trans^ (Figure 1), AUC measurements of tumor and NAWM provided more consistent (K^trans^: 12% failed model fitting; AUC: no missing data) and reproducible values. Mean coefficients of variation of K^trans^ and AUC measurements were similar in tumors (ANOVA *p* = 0.673). In NAWM, the mean coefficients of variation of AUC was significantly less (ANOVA *p* = 0.012) than for K^trans^. As both measurements are recommended endpoints for the assessments and reporting of MRI oncology trials [9], we proceeded to use AUC for subsequent analysis.

**Figure 2:**
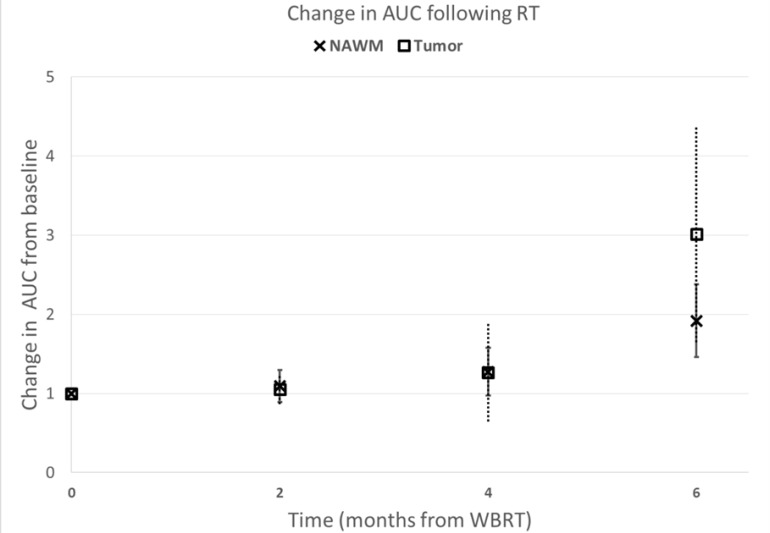
Tumor and normal tissue vascular permeability changes following brain irradiation Contrast uptake following brain irradiation. Increase in contrast uptake (Area Under the uptake Curve (AUC)) of normal appearing white matter (NAWM) and tumor following whole brain irradiation (WBRT). Data were normalized to the AUC at baseline, prior to beginning WBRT. Error bars represent the standard error of the means.

**Figure 3:**
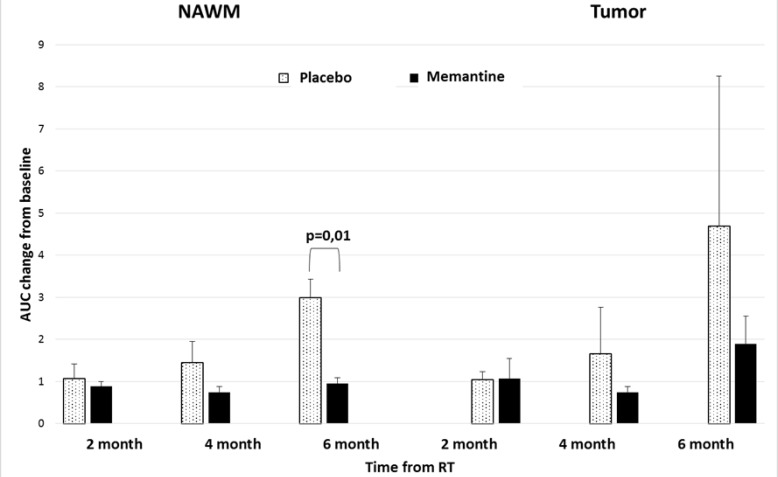
Tissue contrast uptake after irradiation in relation to memantine use Contrast uptake (AUC) of normal appearing white matter (NAWM) and tumor in patients on placebo and memantine. NAWM of patients receiving memantine have reduced AUC changes following radiotherapy (*p* = 0.01) in comparison to patients receiving placebo. Error bars represent the standard error of the means.

We observed a trend to suggest increased AUC of tumors (*p* = 0.09) and NAWM (*p* = 0.06) 6 months following WBRT initiation (Figure 2). We found that patients receiving memantine had significantly (*p* = 0.01) less NAWM AUC changes following radiotherapy than those who received placebo (Figure 3). When the 12 RTOG 0614 patients were analyzed separately, the memantine arm maintained a trend (*p* = 0.03) in the reduction in NAWM AUC changes following radiation as compared to the placebo arm.

**Figure 4:**
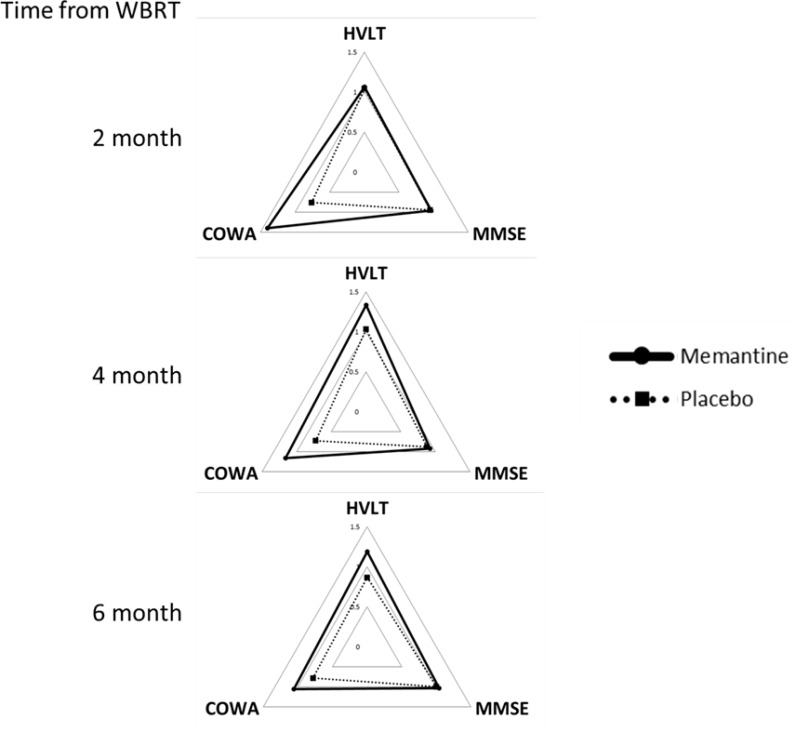
Neurocognitive functions following brain irradiation Neurocognitive functions following brain irradiation. Neurocognitive functions (HVTL, MMSE, and COWA) of patients at 2, 4 and 6 months from receiving whole brain radiotherapy (WBRT) with memantine (solid) or placebo (dotted). Data were normalized to individual patients' baseline values. Higher values represent better quality of life or neurocognitive functions; values > 1 represents an improvement from baseline.

Consistent with the overall results from RTOG 0614, patients on memantine retained better cognitive functions (COWA *p* = 0.03) than those on placebo (Figure 4). HVLT (*p* = 0.10) measures were trending towards improvements in the memantine group as well. The cognitive results and their statistical significance remained the same when the analyses were repeated using RTOG 0614 patients only. QOL data were not compared as only 4 patients who had sequential MRIs completed more than 1 QOL questionnaire. Using blood samples collected prior to each MRIs, we explored the association between CEC and DCE-MRI AUC changes (Supplementary Figure 2). No association was observed between the level of CEC and DCE-MRI changes, time from radiotherapy or memantine use.

## DISCUSSION

In comparison to previous trials that examined the ability of DCE-MRI to detect BBB permeability changes following high-dose radiotherapy targeted at tumors, the current pilot study demonstrated that modest dose WBRT increases the vascular permeability in normal brain tissue (mean NAWM AUC increase from baseline: 1.9 fold; *p* = 0.06). Using an α/β = 2Gy for late glial cell toxicities [10], the mean normalized 2Gy equivalent biologically effective dose (nBED 2/2) of 37.5Gy given in 15 fractions is nBED2/2 = 42.2Gy. Our observation corroborated a previous publication that suggested that neurovascular permeability was increased following a threshold dose of 20Gy given in 30 fractions (nBED2/2 = 13.35Gy) [7]. Although the authors also observed a recovery in the vascular permeability of the brain parenchyma 6 months after radiotherapy, our placebo patients did not show any recovery in their NAWM AUC within the first 6 months after WBRT (Figure 3), suggesting a potential dose related effect to brain vasculature damage and recovery. Similarly, tumor contrast uptake increased over the first 6 months from WBRT.

The detrimental effect of brain radiotherapy is well documented, particularly in studies involving the treatment of childhood cancers [11]. In childhood cancer survivors, the neurocognitive morbidity of radiotherapy is associated with higher radiation dose, larger volume of irradiation, and younger age at the time of treatment. In adult patients, multiple studies examined the consequences of prophylactic cranial irradiation to patient reported QOL. Using EORTC QOL questionnaires, Slotman et al. and Le Pechoux et al. did not detect significant differences in self-reported cognitive functions between patients who received 25Gy/10 fractions (nBED2/2 = 28.13Gy) *vs*. observation [12] or 25Gy/10 fractions *vs*. 36Gy/18-24 fractions [13] (nBED2/2 = 31.5-36Gy), respectively. However, when outcomes were evaluated using standardized neurocognitive tests, Welzel et al. documented acute cognitive dysfunctions soon after WBRT is initiated that remained significant 2-4 weeks after the completion of WBRT [14]. Wolfson et al. similarly found radiation dose and age dependence in the development of neurocognitive defects [15]. Finally, Sun et al. found that in comparison with patients who were observed, patients who received prophylactic cranial irradiation (30Gy in 15 fractions; nBED2/2 = 30Gy) developed significant declines in immediate and delayed recall beginning at 3 months post-WBRT, with mild and incomplete recovery in the following months [16].

The mechanism by which radiation induces brain injury is likely secondary to injuries to multiple cell types, including vascular [17] and parenchymal cells such as neuronal and glial cells [6]. Radiation may induce vascular damage, which leads to ischemia that subsequently results in NMDA receptor stimulation and excitotoxicity. Memantine is a NMDA receptor competitive antagonist that is approved for the treatment of Alzheimer's disease and has shown mixed efficacies in the treatment of vascular dementia. The RTOG 0614 study demonstrated that starting at 8 weeks from WBRT, patients on memantine had less cognitive decline than the placebo cohort [5]. In our study, we observed that patients on memantine were also protected from radiation induced NAWM vasculature permeabilization (Figures 3–4). Our findings were in accordance with another preliminary study that suggested that DCE-MRI could be an imaging biomarker of changes in neurovasculature permeability and increased permeability following radiotherapy potentially predicted for the development of neurocognitive dysfunction following radiotherapy [7]. Similarly, DCE-MRI may be used to detect subtle BBB permeability changes in early neurodegenerative diseases such as Alzheimer's disease [18–20]. Furthermore, a pre-clinical study suggested that memantine reduced brain edema, BBB permeability, infarct volume and neurological deficits when it is given 15 min following middle cerebral artery occlusion in rats [21]. These prior studies corroborate with our results that suggested that memantine not only protected neurons from radiation-induced excitotoxicity, but might also protect the cerebral vasculature from radiation damages.

As shown in Supplementary Figure 3 and 4, the genes (GRIN1, GRIN2A, GRIN2B, GRIN2C, and GRIN2D) and protein of NMDA receptors subunits are widely expressed in different normal tissues and cells, including endothelial cells. Preclinical studies suggested that stimulation of cerebral endothelial cell NMDA receptors could induce vasodilation of brain arteries or excitotoxicity and breakdown of the BBB [22]. Therefore, the neuroprotective effects of memantine might be secondary to the combined reduction in neuronal and endothelial cell deaths from irradiation.

In our study, there was no significant (*p* = 0.20) difference in overall survival of patients on memantine or placebo. There was no significant correlation between the vascular permeability of tumors or NAWM at any time point with overall survival. The majority (78.6%) of our patients had lung or breast cancers, which is in keeping with the patient population in RTOG 0614 (84.6% lung and breast cancers). Results from the RTOG 0614 [5] trial demonstrated that patients who received memantine (*n* = 256) or placebo (*n* = 252) had similar median progression free survival (4.7 *vs*. 5.5 months; *p* = 0.27) and overall survival (6.7 *vs*. 7.8 months; *p* = 0.28). There was also no additional toxicity observed among patients who received memantine *vs*. placebo. Therefore, use of memantine and its neurovascular protective effect did not seem to affect tumor control, patient survival or toxicities.

Due to the small number of patients in this pilot study, our results need to be confirmed using a larger cohort of patients. Also, data from this small patient cohort did not allow us to determine whether surgery and/or radiosurgery influenced the vascular permeability of tumors. Our imaging methodologies could be modified to study the radiotherapy effects to specific brain structures, such as the hippocampus by increasing the spatial resolution of the DCE-MRI acquisition at this structure and by using a power injector. As the gene and protein expressions of different NMDA receptors vary between brain structures (Supplementary Figure 3 and 4), the benefit of memantine may differ between tissues and cell types [23, 24]. Furthermore, as many long-term toxicities secondary to radiation therapy are related to the de-vascularization of normal tissues, the use of memantine as an adjunct to radiotherapy may reduce extra-cerebral endothelial cell injuries and the rate of chronic radiation side-effects in a broad range of patients.

In conclusion, DCE-MRI represents an established functional imaging method employed in evaluating vascular permeability changes in tissues and tumors. Radiation therapy induces vasculature damages and many long-term toxicities are related to the de-vascularization of normal tissues. Our pilot study suggests that DCE-MRI could detect BBB vascular damages secondary to WBRT and could be used as a biomarker of neurocognitive dysfunction. Our study also suggested that memantine reduced WBRT damages to neurons and endothelial cells. Future studies should aim at confirming the usefulness of DCE-MRI as a clinical imaging biomarker of tissue toxicity from radiotherapy. The use of memantine as a potential radioprotector for vascular tissues may be explored to reduce radiation induced side-effects.

## PATIENTS AND METHODS

### Patients

Fourteen adult patients with a pathologically proven diagnosis of solid malignancy within 5 years of registration and with brain metastases visible on contrast-enhanced MRI were recruited to the study. One patient died before WBRT was started. Eligibility criteria included a Karnofsky performance status of ≥ 70, stable systemic disease in the 3 months prior to study entry, serum creatinine ≤ 3 mg/dL, creatinine clearance ≥ 30 mL/min, total bilirubin ≤ 2.5 mg/dL, blood urea nitrogen (BUN) ≤ 20 mg/dL, Mini Mental State Exam (MMSE) score ≥ 18 within 28 days of study entry, no current alcohol or drug abuse, no chronic use of benzodiazepines, and no severe active comorbidity. Patients could have received prior therapy for brain metastasis, including radiosurgery and surgical resection (but no prior cranial external beam radiotherapy). Patients receiving systemic therapy were eligible if such therapy was given > 14 days prior to study entry, and they could not receive chemotherapy for at least 14 days after completing radiotherapy. Table 1 summarizes the patients' characteristics. The study was approved by the McGill University Health Centre Research Ethics Board. All patients provided written informed consent.

### Treatment

All 12 patients concurrently randomized on RTOG 0614 were treated with WBRT consisting of 37.5 Gy in 15 fractions, delivered using 2 parallel-opposed fields with or without subfields. Of the 2 patients not on RTOG 0614, patient #3 received 30 Gy in 12 fractions and patient #10 received 37.5 Gy in 15 fractions, delivered using 2 parallel-opposed fields with or without subfields (Table 1). Patients enrolled in the RTOG 0614 trial were randomly assigned to received placebo (*n* = 5) or memantine (*n* = 7) at the initiation of WBRT. Patient on memantine received 5mg on week 1, increasing by 5mg every week until the target dose of 10mg twice daily, and was then maintained at this dose for a total of 24 weeks [5]. Patients not on the RTOG0614 did not receive placebo or memantine; for the purpose of analysis, data from these patients were combined with those who received placebo. Following the completion and publication of final data from the RTOG 0614 study, we obtained permission from the RTOG to unblind the treatment (placebo or memantine) that patients received during and after WBRT. One of the 14 patients died prior to receiving the first fraction of WBRT.

### Imaging

Patients underwent MRI's prior to WBRT (within 28 days from start of WBRT), then at 8-weeks, 16-weeks and 24-weeks after the initiation of WBRT.

Timing and MRI sequence acquisition were modified from the methods described by Roberts et al. [25]. All images were acquired on a Siemens 3T Tim Trio with a maximum gradient strength of 40mT/m and a slew rate of 200 mT/m/ms, using a Siemens 32-channel head coil.

Briefly, a sagittal T2-weighted scout scan was obtained to plan a whole brain T1-weigthed MPRAGE (matrix: 256×256×176, 1mm^3^ isotropic resolution, TR/TE = 2.3s/3.4ms, FA = 9^0^), localize the tumor, and plan the dynamic series. For DCE-MRI, a 3D-FLASH sequence was obtained with the following parameters: TR/TE = 4.4/1.5ms; flip angle: 25°; matrix: 128 × 128, 22 slices; resolution: 2×2×6 mm^3^; BW = 1775Hz/px,; GRAPPA factor 2. This 3D data set was acquired every 6 secs, starting before intravenous bolus administration of a single dose of contrast material (0.1 mmol/kg Gadovist^®^, Bayer Inc.) and continuing for the subsequent 15 minutes, for a total of 155 dynamic images. The contrast agent was administered manually as a bolus injection and chased with 30 ml of saline. At the completion of 15 minutes of DCE-MRI, a second, post-contrast MPRAGE, matched to the first acquisition, was acquired. All kinetic images and post-contrast MPRAGE were registered to the initial pre-contrast MPRAGE using the MINC software tools (http://packages.bic.mni.mcgill.ca/tgz). Six-parameter linear registration using mutual information was computed between the first dynamic image and the MPRAGE anatomical. Then each image from the dynamic series was registered to the first image using a 6-parameter linear transformation using cross-correlation. The transformations from these two linear registrations were concatenated and applied to each dynamic image so that all frames were registered to the high resolution anatomical.

3D-FLASH images were acquired for purposes of T1-mapping using the DESPOT1 technique (FA = 2°/10°, 128×128×22 matrix, resolution 2×2×6mm3) [26] (Supplementary Figure 1). The T1-weighted time series was used together with the equilibrium magnetization (M0) map calculated with DESPOT1 to generate quantitative T1 4D volumes and were subsequently converted to concentration using the pre-contrast T1 (T10) maps and contrast agent relaxivity in the plasma (values at 3T, 37°C, pH = 7, in plasma: r1(Gd-BT-DO3A) = 5.0±0.3 mM-1s-1 [27–29]).

All regions of interests (ROI) (tumor, NAWM, sagittal sinus) were manually segmented on the post-contrast MPRAGE by PW. The area of tumor was defined as the contrast enhancing volumes (using the pre and post-injection MPRAGE scans) if the tumor was not resected. If multiple tumors were present, each tumor was segmented as an individual ROI. In the case of resected tumors, any contrast-enhancement surrounding the tumor cavity was contoured. A parenchymal area of the brain contralateral to the tumor, without contrast enhancement on post-injection MPRAGE scans, was considered as NAWM. A segment of the superior sagittal sinus was contoured using contrast-enhanced images (3D-FLASH and MPRAGE sequences) and used for measuring the vascular time course of the contrast agent concentration. Although signals from the sagittal sinus are not arterial, the large size of this vein reduces experimental/analytical partial volume effects. This methodology has been shown to be highly reproducible [30, 31] and a good surrogate for the arterial input function [32, 33].

Concentrations over time were computed using a Matlab (Mathworks, Natick, MA) program developed in-house, and the sagittal sinus ROI was used to define the blood input function. The signal intensity changes (signal intensity after contrast agent administration minus that before contrast agent administration) were calculated for the ROIs for each post-injection time point, and their time courses were used for subsequent kinetic analyses.

Vascular permeability was evaluated using the semi-quantitative Initial Area Under the Curve (AUC) Blood Adjusted method [34, 35], which was calculated as the area under the ROI signal intensity curve from the time of injection to 90 seconds post-injection, divided by the area under the blood concentration curve for the same period of time. This methodology provided simple and robust measurements that did not require model fitting and has been recommended as a primary measure in MRI trials of oncology therapeutics [9]. We also explored the traditional kinetic modeling methods described by Tofts et al [36]. Specifically, a bidirectional 3-compartment model based on the equations of Tofts and Kermode [36], which yielded estimates of fractional tissue blood volume (in milliliters per cubic centimeters), plasma volume, and microvascular permeability, which was expressed as the transendothelial transfer constant, K^trans^, was also evaluated. An example of parametric maps of K^trans^, ve and vp and the corresponding K^trans^ of tumor voxels obtained from the Tofts and Kermode modeling are shown in Figure 1.

### Quality of life and neurocognitive assessments

At each MRI time point, cognitive, quality-of-life (QOL) assessment and blood samples were collected according to the patient's ability to tolerate the exams. Cognitive assessments were done using the MMSE, Hopkins Verbal Learning Test (HVLT), and Controlled Oral Word Association Test (COWA). QOL assessments were made using validated questionnaires: FACT-G and FACT-Br.

### Circulating endothelial cell assessments

Circulating endothelial cells (CEC) were measured as per the protocol described by Duda et al. [37]. Briefly, patient blood was collected prior to MRI imaging using tubes containing EDTA. Fluorescently labelled antibodies were purchased from BD Pharmingen: CD31-FITC, CD34-APC, CD133-PE, CD45-PerCP, Fluorescently labeled isotype-matched IgG1 antibodies, and VEGFR2 (KDR)-PE. Cells from the buffy coat were fixed, labeled using the above listed antibodies, then sorted using the FACSCalibur flow cytometer (Becton Dickinson).

### Analysis plan

This study was a prospective pilot study with the aim of recruiting up to 15 patients in order to determine the feasibility and ability of DCE-MRI in detecting vascular permeability changes secondary to WBRT. With no prior evidence of DCE-MRI's capacity to detect WBRT-related NAWM neurovascular changes at the time of the trial design, the study was not pre-designed with statistical power estimations. As Cao et al. observed an initial increase in vascular permeability followed by a gradual recovery after the completion of radiotherapy [7], a linear change in vascular permeability over time could not be inferred; hence linear regression and slope analysis might not be appropriate for our data. Therefore, the analysis consisted of comparing the vascular permeability in tumor and NAWM before *vs*. after WBRT (3 time points within 6 months from the start of WBRT) using the paired student *t*-test to compare baseline data with 2, 4 and 6 months MRI data. Coefficient of variations of DCE-MRI measurements (K^trans^ and AUC) were calculated (*C_v_ = Standard deviation/Mean*) and compared using ANOVA. Comparison between placebo and memantine for MRI and neurocognitive results were made using the student *t*-test at each time point. Changes in CEC levels were analyzed using the spearman correlation test to determine its association with MRI data. All analyses were two-sided and a *P*-value of < 0.05 is considered as statistically significant. The Bonferroni correction was applied to the change in vascular permeability analyses of 3 different time points (2, 4 and 6 months) such that a *P*-value of < 0.017 is considered statistically significant.

## SUPPLEMENTARY MATERIALS FIGURES


