# Supplementary material for: A pilot study using dynamic contrast enhanced-MRI as a response biomarker of the radioprotective effect of memantine in patients receiving whole brain radiotherapy
Source: Oncotarget. 2016 May 28;7(32):50986–96. doi: 10.18632/oncotarget.9653 (PMC5239453; doi:10.18632/oncotarget.9653)
Supplement: Supplementary file 1 [file oncotarget-07-50986-s001.pdf]

A pilot study using dynamic contrast enhanced-MRI as a response biomarker of the radioprotective effect of memantine in patients receiving whole brain radiotherapy

Supplementary Material

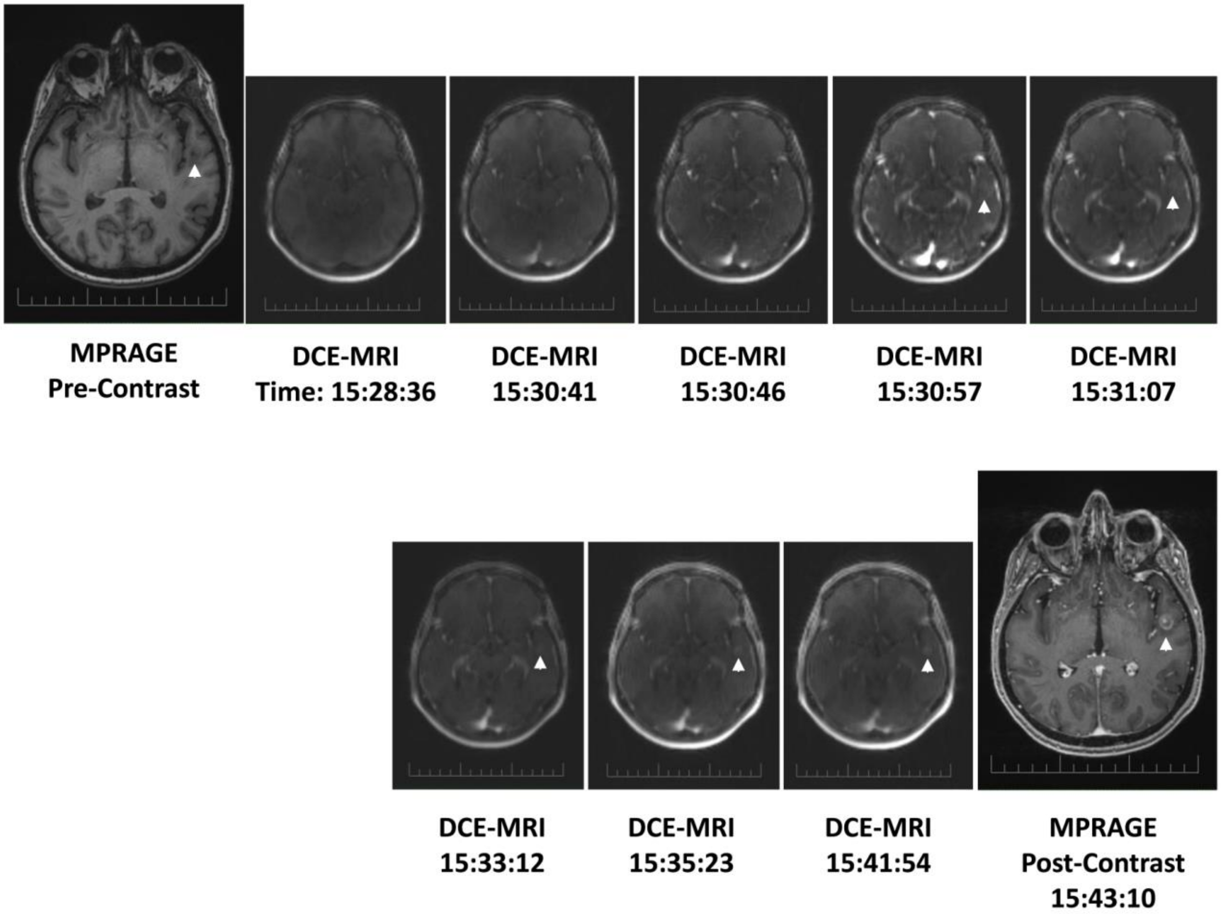

Supplementary Figure 1: Example of dynamic contrast enhanced-MRI images.

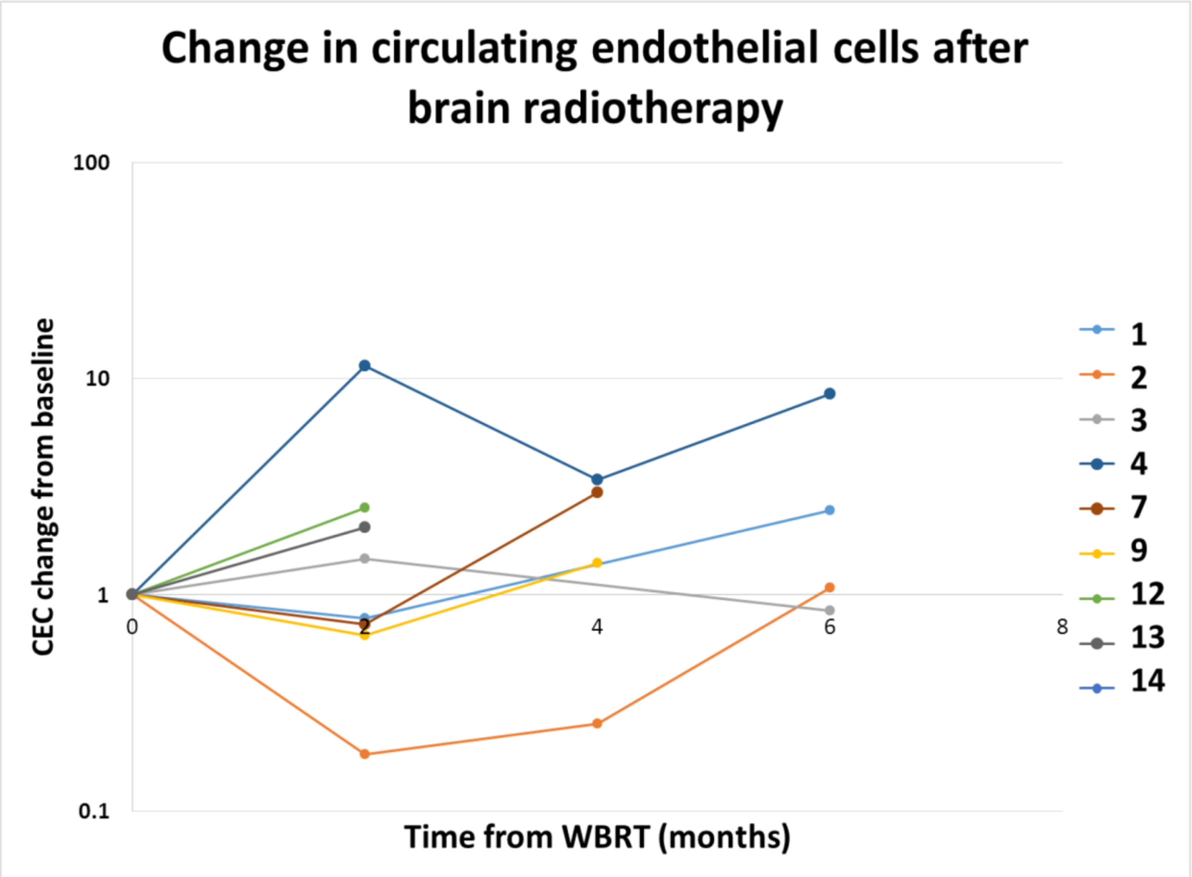

Supplementary Figure 2: Change in circulating endothelial cells (CEC) following whole brain radiotherapy (WBRT). Data was normalized to the baseline level of CEC prior to WBRT.

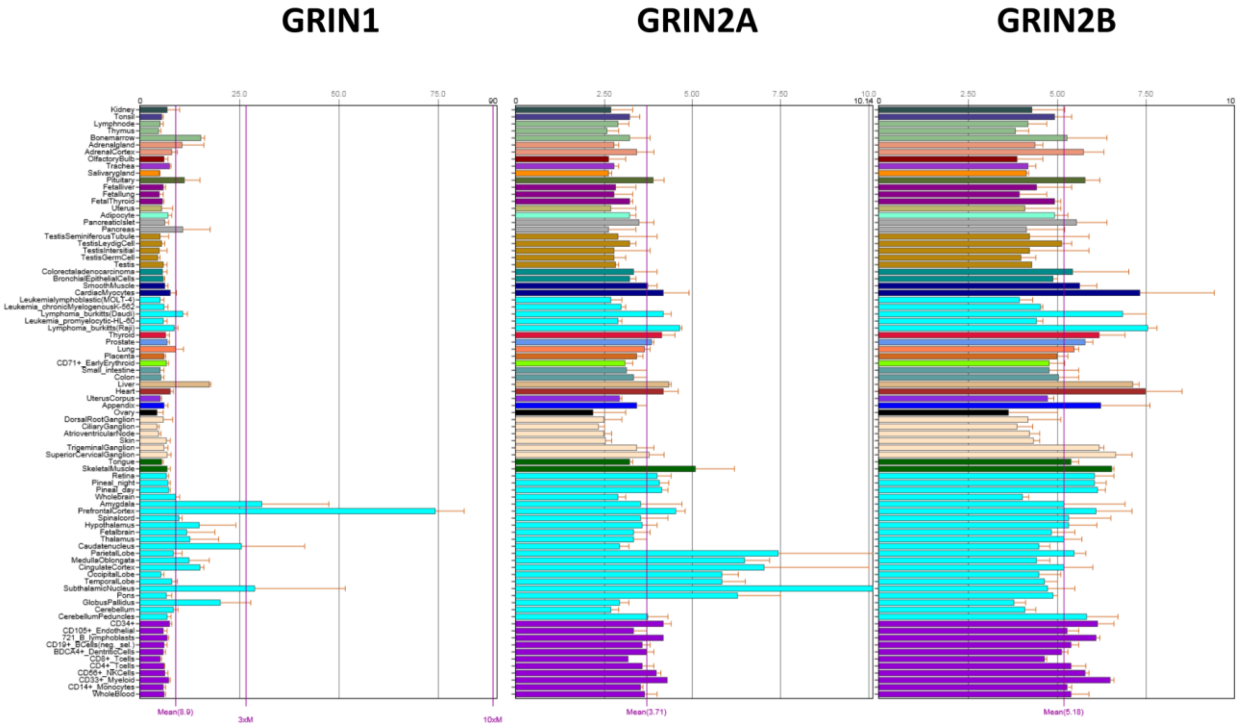

Supplementary Figure 3: Gene expressions of 3 of the NMDA receptor subunits (GRIN1, GRIN2A and GRIN2B) in different human tissues. Data derived from BioGPS Human U133 microarrayGeneAtlas data.

GRIN1

TISSUE | SUBCELL | CELL LINE | CANCER

ANTIBODY STAINING OVERVIEW ? »

Antibodies in assay CAB006831

Tissue presentation order: Organ | Cell type | Alphabetical

Level of antibody staining/expression: High | Medium | Low | Not detected

Tissue		Antibody staining
**Nervous system cells**		
Soft tissue 1	Peripheral nerve	Not detected
Soft tissue 2	Peripheral nerve	
Cerebral cortex	Neuronal cells	High
Hippocampus	Neuronal cells	High
Lateral ventricle	Neuronal cells	High
Cerebellum	Purkinje cells	High
Cerebellum	Cells in granular layer	High
Cerebellum	Cells in molecular layer	High
Colon	Peripheral nerve/ganglion	High
Cerebral cortex	Glial cells	High
Hippocampus	Glial cells	High
Lateral ventricle	Glial cells	Not detected
Cerebral cortex	Neuropil	Medium
**Hematopoetic cells**		
Skin 1	Langerhans	Medium
Bone marrow	Hematopoietic cells	Medium
Lymph node	Germinal center cells	Medium
Tonsil	Germinal center cells	Medium
Lymph node	Non-germinal center cells	High
Tonsil	Non-germinal center cells	High
Spleen	Cells in white pulp	Medium
Spleen	Cells in red pulp	Medium
Appendix	Lymphoid tissue	Low
Lung	Macrophages	Medium
**Glandular and transitional epithelial cells**		
Salivary gland	Glandular cells	Low
Stomach 1	Glandular cells	Medium
Stomach 2	Glandular cells	Medium
Duodenum	Glandular cells	Medium
Small intestine	Glandular cells	Medium
Appendix	Glandular cells	Medium
Colon	Glandular cells	Medium
Rectum	Glandular cells	High
Liver	Bile duct cells	Not detected
Gallbladder	Glandular cells	Medium
Pancreas	Exocrine glandular cells	Medium
Breast	Glandular cells	Medium
Cervix, uterine	Glandular cells	Not detected
Endometrium 1	Glandular cells	Medium
Endometrium 2	Glandular cells	Low
Fallopian tube	Glandular cells	High
Epididymis	Glandular cells	Low
Prostate	Glandular cells	Low
Seminal vesicle	Glandular cells	Not detected
Bronchus	Respiratory epithelial cells	Medium
Nasopharynx	Respiratory epithelial cells	Medium
Urinary bladder	Urothelial cells	Medium

CAB006831

Tissue		Antibody staining
**Squamous epithelial cells**		
Skin 1	Keratinocytes	Medium
Skin 2	Epidermal cells	High
Vagina	Squamous epithelial cells	Medium
Cervix, uterine	Squamous epithelial cells	Medium
Oral mucosa	Squamous epithelial cells	Medium
Tonsil	Squamous epithelial cells	High
Esophagus	Squamous epithelial cells	Medium
**Endocrine cells**		
Pancreas	Islets of Langerhans	Medium
Thyroid gland	Glandular cells	Medium
Parathyroid gland	Glandular cells	Low
Adrenal gland	Glandular cells	Medium
Testis	Leydig cells	Medium
**Mesenchymal cells**		
Soft tissue 1	Adipocytes	Not detected
Soft tissue 1	Chondrocytes	Not detected
Soft tissue 1	Fibroblasts	Medium
Soft tissue 2	Fibroblasts	
Soft tissue 2	Adipocytes	Not detected
Soft tissue 2	Chondrocytes	
Skin 1	Fibroblasts	Medium
Skeletal muscle	Myocytes	Not detected
Heart muscle	Myocytes	Not detected
Smooth muscle	Smooth muscle cells	Not detected
Endometrium 1	Cells in endometrial stroma	Low
Endometrium 2	Cells in endometrial stroma	Not detected
Ovary	Ovarian stroma cells	Low
Breast	Adipocytes	Not detected
**Other cells**		
Liver	Hepatocytes	Not detected
Skin 1	Melanocytes	Medium
Kidney	Cells in glomeruli	Medium
Kidney	Cells in tubules	Medium
Lung	Pneumocytes	Not detected
Testis	Cells in seminiferous ducts	High
Ovary	Follicle cells	
Colon	Endothelial cells	Medium
Placenta	Decidual cells	Medium
Cerebral cortex	Endothelial cells	Not detected
Placenta	Trophoblastic cells	Medium
Breast	Myoepithelial cells	Not detected

CAB006831

Supplementary Figure 4: NMDA receptor subunit 1 protein expression in different human tissues and cells. Data derived from The Human Protein Atlas.
